# Isolation and Characterization of Activators of ERK/MAPK from *Citrus* Plants

**DOI:** 10.3390/ijms13021832

**Published:** 2012-02-09

**Authors:** Yoshiko Furukawa, Satoshi Okuyama, Yoshiaki Amakura, Sono Watanabe, Takahiro Fukata, Mitsunari Nakajima, Morio Yoshimura, Takashi Yoshida

**Affiliations:** 1 Department of Pharmaceutical Pharmacology, College of Pharmaceutical Sciences, Matsuyama University, 4-2 Bunkyo-cho, Matsuyama, Ehime 790-8578, Japan; E-Mails: sokuyama@cc.matsuyama-u.ac.jp (S.O.); watanabe.sono.mm@ehime-u.ac.jp (S.W.); 16061157@cc.matsuyama-u.ac.jp (T.F.); mnakajim@cc.matsuyama-u.ac.jp (M.N.); 2 Department of Pharmacognosy, College of Pharmaceutical Sciences, Matsuyama University, 4-2 Bunkyo-cho, Matsuyama, Ehime 790-8578, Japan; E-Mails: amakura@cc.matsuyama-u.ac.jp (Y.A.); myoshimu@cc.matsuyama-u.ac.jp (M.Y.); tyoshida@cc.matsuyama-u.ac.jp (T.Y.)

**Keywords:** methoxyflavone, 3,5,6,7,8,3′,4′-heptamethoxyflavone, ERK1/2, *Citrus grandis*, Kawachi bankan, neurons, MK-801

## Abstract

Extracellular signal-regulated kinases 1/2 (ERK1/2), components of the mitogen-activated protein kinase (MAPK) signaling cascade, have been recently shown to be involved in synaptic plasticity and in the development of long-term memory in the central nervous system (CNS). We therefore examined the ability of *Citrus* compounds to activate ERK1/2 in cultured rat cortical neurons, whose activation might have a protective effect against neurodegenerative neurological disorders. Among the samples tested, extracts prepared from the peels of *Citrus grandis* (Kawachi bankan) were found to have the greatest ability to activate ERK1/2. The active substances were isolated by chromatographic separation, and one of them was identified to be 3,5,6,7,8,3′,4′-heptamethoxyflavone (HMF). HMF significantly induced the phosphorylation of cAMP response element-binding protein (CREB), a downstream target of activated ERK1/2, which appears to be a critical step in the signaling cascade for the structural changes underlying the development of long-term potentiation (LTP). In addition, the administration of HMF into mice treated with NMDA receptor antagonist MK-801 restored the MK-801-induced deterioration of spatial learning performance in the Morris mater-maze task. Taken together, these results suggest that HMF is a neurotrophic agent for treating patients with memory disorders.

## 1. Introduction

Evidence has begun to accumulate that indicates health-promoting effects of low-molecular weight and non-nutrient phytochemicals on human beings. Numerous quantitative studies have been conducted on flavonoids among various phytochemicals and indicate that flavonoids have anti-tumor, anti-inflammatory, and anti-oxidant ability as well as beneficial cardiovascular properties [1,2]. Some recent studies have demonstrated that flavonoids are also able to exert powerful actions on mammalian cognition and may reverse age-related declines in memory and learning [3]. For example, fisetin (3,7,3′,4′-tetrahydroxyflavone), which can be found in many plants, where it serves as a coloring agent, was reported to facilitate long-term potentiation (LTP) in rat hippocampal slices, and to enhance object recognition in mice [4]. Nobiletin (5,6,7,8,3′,4′-hexamethoxy flavone; NBT), one of the *Citrus* polymethoxyflavones especially present in *Citrus unshiu* and *Citrus depressa* Hay, has been shown to have a neuroprotective effect in the central nervous system (CNS); *i.e.*, it has the ability to rescue rodents from Aβ-induced impairment of learning ability [5,6], bulbectomy-induced cholinergic neurodegeneration [7,8], and ischemia-induced learning and memory deficits [9].

*Citrus* fruits, which are cultivated all over the world, are known to be a rich source of flavonoids, some of which are unique to *Citrus* plants [10]. Thus we decided to search for health-benefiting flavonoids in representative *Citrus* species grown in Ehime Prefecture of Japan, which is an eminent citrus-growing district in Japan. As test samples, we prepared ethanol extracts from the peels of green unripe fruits.

For the screening method, we analyzed the ability of these ethanol extracts to activate (cause the phosphorylation of) extracellular signal-regulated kinase (ERK) 1/2 of cultured neurons. This parameter was chosen because, ERK1/2, one of the most common signal transduction molecules by which extracellular stimuli are propagated from the cell surface to cytoplasmic and nuclear effectors, has been shown to be involved in synaptic plasticity and in the development of LTP in the CNS [11]. In the present study, we successfully identified 3,5,6,7,8,3′,4′-heptamethoxyflavone (HMF) as one of the *Citrus* substances that induce the activation of ERK1/2.

ERK1/2 activation is known to lead to a number of cellular changes associated with the development of long-term memory, such as the expression of the cAMP response element-binding protein (CREB) [12], which is a transcription factor located within the nucleus. CREB activation (phosphorylation) appears to be a critical step in the signaling cascade that leads to the structural changes underlying the development of LTP [13]. Therefore, we also examined whether HMF could stimulate the phosphorylation of CREB in cultured neurons. Furthermore, we studied whether subcutaneous injection of HMF could rescue mice from drug-induced learning impairment.

## 2. Materials and Methods

### 2.1. Preparation of Extracts from *Citrus* Species

All *Citrus* fruits examined, 8 *Citrus* cultivers including tangor, mandarin, and orange groups, were harvested when they were still green from trees in the same field (Ehime Mandarin Research Center, Uwajima, Ehime, Japan) and same year (2008). Each fresh peel of *Citrus* fruits (100 g) was cut into small pieces and extracted with ethanol (*ca*. 500 mL) at room temperature. The extracts were concentrated *in vacuo* below 40 °C, and each extract was then tested in biological assays.

### 2.2. Preparation of Extracts of *Citrus Grandis* (Kawachi bankan)

Fresh peels (110 g) of *C. grandis* were homogenized in ethanol (600 mL). The filtered homogenate was concentrated *in vacuo* below 40 °C to give an ethanol extract (7.8 g). A part (5.0 g) of the ethanol extract was dissolved in water (50 mL), and successively extracted with *n*-hexane (50 mL × 5) and ethyl acetate (50 mL × 5) to yield *n*-hexane (285.3 mg), ethyl acetate (461.0 mg), and water (4.1 g) extracts. The extracts were concentrated *in vacuo* below 40 °C, and each extract was then subjected to biological assays.

### 2.3. Analysis of *n*-hexane Extracts of *Citrus Grandis*

The *n*-hexane extract (225 mg) was applied to a chromatography column containing silica gel (Nacalai Tesque, Kyoto, Japan: 75 μm, 1.5 i.d. × 40 cm) and eluted with *n*-hexane containing increasing proportions of ethyl acetate to give HMF (8.5 mg), together with auraptene (AUR; 39 mg), tangeretin (5,6,7,8,4′-pentamethoxyflavone, TGN; 2.7 mg), and NBT (1.1 mg). The compounds were identified by comparison with their spectral data reported in the literature. The NMR and MS data on HMF, which were determined with Brucker AVANCE500 and Brucker micrOTOF-Q instruments, respectively, were the following: ^1^H NMR (500 MHz, CDCl_3_) *δ* 7.83 (1H, d, *J* = 2.0 Hz, H-2′), 7.80 (1H, dd, *J* = 2.0, 9.5 Hz, H-6′), 7.00 (1H, d, *J* = 9.5 Hz, H-5′), 4.08, 3.99, 3.958, 3.957, 3.93, 3.87 (each 3H, s, –OCH_3_). ^13^C NMR (126 MHz, CDCl_3_) *δ* 151.1 (C-2), 140.9 (C-3), 174.0 (C-4), 144.0 (C-5), 138.0 (2C, C-6, 8), 151.4 (C-7), 148.3 (C-9), 115.2 (C-10), 123.6 (C-1′), 111.1 (C-2′), 148.9 (C-3′), 153.2 (C-4′), 111.2 (C-5′), 122.1 (C-6′), 62.4, 62.0, 61.9, 61.7, 59.9, 56.1, 56.0 (–OCH_3_). ESI-MS *m/z* 433 [M + H]^+^.

### 2.4. Preparation of HMF from Orange Oil

Commercial orange oil [500 mL; Wako (Osaka, Japan)] was applied to a silica gel column (Nacalai Tesque; 75 μm, 5.0 i.d. × 67 cm) and eluted with *n*-hexane containing increasing amounts of ethyl acetate to provide HMF (944.8 mg).

### 2.5. Cell Cultures and Reagents

For the preparation of neuron cultures, the neocortices of 18-day-old embryonic Wistar rats (Charles River Japan, Inc., Yokohama, Japan) were dissected out; treated with phosphate-buffered saline (PBS) containing 0.25% trypsin, 10 mM glucose, and DNAase (6 μg/mL, Sigma, St. Louis, MO, USA); and mechanically dissociated. Following centrifugation (900 × g, 3 min), the cell pellet was resuspended in Dulbecco’s modified Eagle’s medium (Sigma) containing 5% fetal bovine serum (ICN Biochemicals, Aurora, OH, USA); and the resuspended cells were plated in culture dishes precoated with poly dl-ornithine (Sigma). After a 24-h culture period, the medium was changed to Neurobasal medium (Invitrogen, Carlsbad, CA, USA) containing B27 supplement (Invitrogen), 2 mM glutamine; and the cells were then cultured for 3 days. Brain-derived neurotrophic factor (BDNF) was purchased from Pepro Tech. (Rocky Hill, NJ, USA). AUR, NBT, and TGN used for assay were purchased from Wako. U0126 and PD98059 were obtained from Wako and Sigma, respectively.

### 2.6. Immunoblot Analysis

Cells in 6-well plates (10^5^ cells/cm^2^) were incubated with test compounds for 30 min, and the cell extracts were prepared as previously described [14]. Proteins (20 μg) in each cell extract were separated on an SDS-polyacrylamide gel and electroblotted onto an Immuno-Blot^TM^ PVDF Membrane (BIO-RAD, Hercules, CA, USA). The primary antibodies used in immunoblotting analysis were rabbit antibody against MAPK 1/2 (Erk1/2-CT), which recognizes the *C*-terminal 35 amino acids of the rat 44-kDa MAPK1/ERK1 and 42-kDa MAPK2/ERK2 (Upstate, Lake Placid, NY, USA); rabbit antibody against phospho-p44/42 MAPK (Thr202/Tyr204), which recognizes phosphorylated ERK1/2 (pERK1/2); and rabbit antibodies against CREB and phosphorylated CREB (Ser-133; Cell Signaling, Woburn, MA, USA). The secondary antibody was horseradish peroxidase (HRP)-linked anti-rabbit IgG (Cell Signaling). The blots were developed by use of the chemiluminescence method with Plus Western Blotting Detection Reagents (Amersham, Piscataway, NJ, USA).

### 2.7. MTT Assay

Cells were plated in 96-well plates, and the MTT assay was performed as described previously [14].

### 2.8. Animals and Drugs

Eight-week-old male C57BL/6 mice (Japan SLC, Shizuoka, Japan) were used. The mice were housed under conditions of controlled temperature and humidity (23 ± 1 °C and 50 ± 10%) on a 12-h light/12-h dark cycle with *ad libitum* access to food and water. HMF was delivered through Alzet osmotic minipumps (DURECT Corp., Cupertino, CA, USA) implanted subcutaneously in the back of the animals. The actual concentration of HMF for pump delivery was calculated on a weight basis, in such a way as to obtain a steady release of 50 mg/kg/day for 7 days. Control animals received the HMF vehicle (DMSO/PEG300, 1:1). NMDA receptor antagonist MK-801 (Sigma) diluted in saline was intraperitoneally (i.p.) injected at the concentration of 0.05 mg/kg, 30 min before behavior test. For sham-operated mice, saline was injected. All experiments were performed in accordance with the Guide Lines for Animals Experimentation prepared by Animal Care and Use Committee of Matsuyama University.

### 2.9. Morris-Type Water Maze (MWM) Test

We conducted the MWM test [15] using modifications as described below. The water maze pool was a circular tank of 110 cm in diameter and 60 cm in depth. The pool was filled with tap water (24–25 °C) and a 10-cm diameter round-shaped platform was hidden at a fixed position 1.5 cm under the surface of water. Training sessions were carried on 3–6 days after pump implantation. Each mouse performed 5 trials per day. We defined a successful escape, *i.e.*, standing on the platform, as a stop for more than 5 s with all limbs on the platform. The cut-off time in a trial was set at 60 s. When the mouse failed to reach the platform in 60 s, it was picked from the water and placed on the platform for 10 s to memorize the location. In such a case, the time needed to escape to the platform (escape latency) was recorded as 60 s. The interval of each trial was 15 min. Probe test was conducted 7 days after the implantation of the osmotic pump, during which the mice swam for 60 s in the absence of the platform. The water maze activity was analyzed by using a video-tracking system, ANY-Maze (Stoelting, Wood Dale, IL, USA). Five mice were used in each group.

## 3. Results and Discussion

### 3.1. ERK1/2 Activation in Neuron Cultures by *Citrus* Extracts

Ethanol extracts of *Citrus* species were prepared by the homogenization of the *Citrus* peels in 5 volumes of ethanol followed by the concentration *in vacuo* to put away ethanol. The powdered ethanol extracts were then dissolved in dimethyl sulfoxide (DMSO) at the concentration of 100 mg/mL, and its required amount (one thousandth) for a final concentration (100 μg/mL) was added to the medium, as it is well known that various cells are not influenced by DMSO at the concentration of 0.1%.

When the neurons were cultured for 30 min in the presence of each ethanol extract prepared from various *Citrus* peel, most of them showed the ability to phosphorylate ERK1/2, but the activity of the extract of *Citrus grandis* (Kawachi bankan) was higher than that of the other citrus extracts (Table 1).

Table 1 also shows that 0.1% DMSO had no effect on the phosphorylation of ERK1/2. The MTT assay showed that the cell viability was unchanged when the cells were treated with 100 μg/mL of a given extract (data not shown).

**Figure 1:**
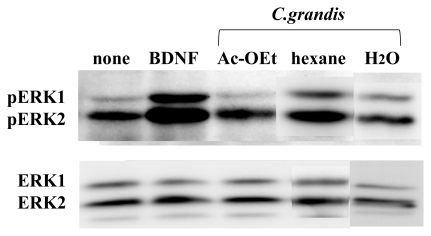
Effects of ethyl acetate (Ac-OEt), *n*-hexane and H_2_O (H_2_O) extracts of *Citrus grandis* (Kawachi bankan) on ERK1/2 activation in rat cortical neurons. Cells were treated with 100 μg/mL of each extract for 30 min or 50 ng/mL brain-derived neurotrophic factor (BDNF) for 10 min, and then equal amounts of protein were analyzed by immnoblot analysis.

The ethanol extract of *C. grandis* (Kawachi bankan) was further partitioned into *n*-hexane, ethyl acetate, and water-soluble portions. Each extractive portion was evaporated to dryness *in vacuo* and then dissolved in DMSO at the concentration of 100 mg/mL. When the cells were treated with 100 μg/mL of each extract for 30 min, only the *n*-hexane extract had the ability to phosphorylate ERK1/2 (Figure 1). These results suggest that some non-polar compound(s) of *C. grandis* (Kawachi bankan) had this ability.

### 3.2. Identification of the Components from *Citrus grandis* Peel

**Figure 2:**
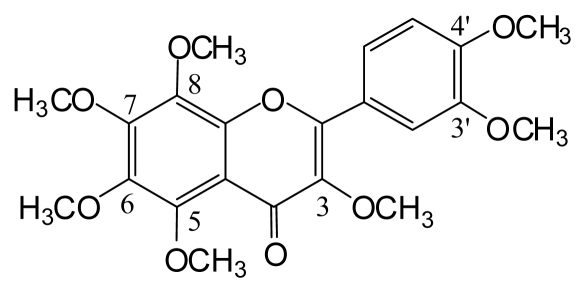
Chemical structure of 3,5,6,7,8,3′,4′-heptamethoxyflavone (HMF).

We then isolated and characterized the active compound(s) in the hexane extract of *C. grandis*. Chromatographic separation of the hexane extract revealed 4 known compounds, which were characterized by spectroscopic analysis as a coumarin derivative with a monoterpene unit (AUR) and 3 polymethoxyflavones (TGN, NBT, and HMF; Figure 2).

The biological actions of TGN and NBT have been vigorously investigated in recent years [3], including their anti-inflammatory activity [16], anti-neuroinflammatory activity [17], and anti-tumor activity [18]; and these compounds have been shown to improve impaired memory loss [5–9], and to have an antidepressant-like effect [19] in the case of NBT. In contrast, HMF has been studied little biologically except for its anti-bacterial [20], anti-tumor [21], and anti-inflammatory [22] activities in peripheral tissues. As there are no reports showing a relationship between HMF and brain function, we decided next to examine the effect of HMF on cultured neurons. As for AUR, its anti-inflammatory activity in the peripheral tissues has been extensively studied [23,24].

### 3.3. HMF-Mediated ERK1/2 Activation in Neuron Cultures

We obtained HMF in sufficient amount for evaluation of its biological potency from commercially available orange oil, as described in Materials and Methods, and examined its ability to cause the phosphorylation of ERK1/2 in cultured neurons.

**Figure 3:**
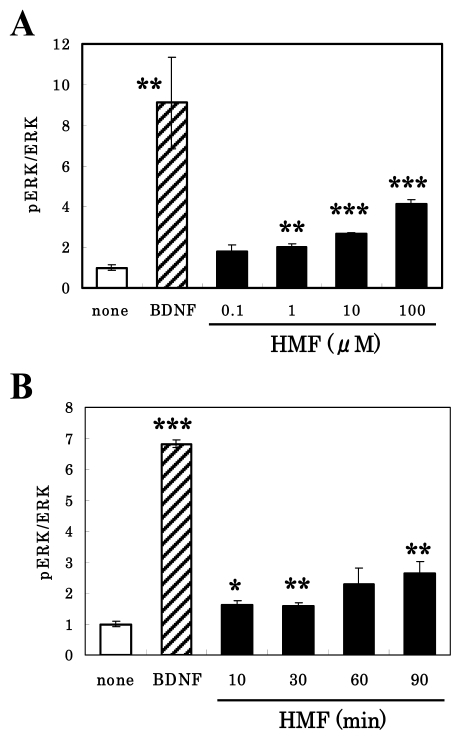
Time- and dose-dependent phosphorylation of ERK2 after HMF treatment of cultured rat cortical neurons. (**A**) The cells were treated for 30 min with various concentrations (0, 0.1, 1.0, 10, and 100 μM) of HMF or BDNF (50 ng/mL) for 10 min, and cell lysates were then prepared and applied to immunoblot analysis; (**B**) Cells were treated with 100 μM HMF for various times (0, 10, 30, 60, and 90 min) or with BDNF (50 ng/mL) for 10 min, and the cell lysates were then prepared for immunoblot analysis. The density ratio of pERK2 to total ERK2 in untreated cultures was expressed as 1 arbitrary unit. Results represent mean ± SEM (*n* = 4, different cultures). Significant difference in values between the compound-treated and non-treated cells: * *P* < 0.05; ** *P* < 0.01; *** *P* < 0.01 (Student’s *t* test).

At first, we performed a dose-response experiment using the purified HMF and cultured neurons. Although neurons have both ERK1 and ERK2, and both of them are phosphorylated by BDNF or *Citrus* compounds (Figure 1), only the ERK2 isoform has been suggested to be attributable to neurogenesis and cognitive function [25]. We therefore analyzed the ratio of phosphorylated ERK2 (pERK2) to total ERK2 (ERK2) hereafter. As shown in Figure 3A, the phosphorylation of ERK2 in neurons occurred in a dose-dependent manner when the cells were cultured with various concentrations (0, 0.1, 1.0, 10, and 100 μM) of HMF for 30 min. Significant toxicity was not detected even at the concentration of 100 μM, as evidenced by the results of the MTT assay (data not shown).

We then examined the pERK2/ERK2 levels in cultured neurons when the cells were cultured for various times (0, 30, 60, and 90 min) in medium containing 100 μM HMF. Enhancement began at 10 min, and the signal was strengthened until 90 min after the start of HMF exposure (Figure 3B). In contrast, BDNF caused a rapid phosphorylation of ERK2 within 10 min (hatched bar). These observations demonstrate that HMF caused the phosphorylation of ERK1/2 with a time-course different from that of BDNF.

**Figure 4:**
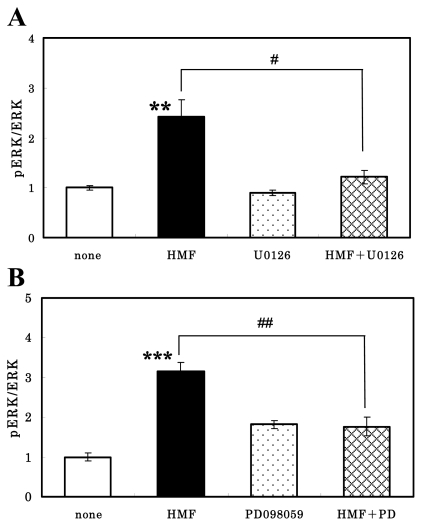
Effects of MEK inhibitors on HMF-induced ERK2 activation in cultured rat cortical neurons. Cells were sequentially pretreated for 30 min with 10 μM PD980059 (**A**) or 10 μM U0126 (**B**) and then incubated for 30 min with or without 50 μM HMF. Thereafter they were subjected to the immunoblot analysis. The ratios of the value for the drug-treated cells to that value for the control cells were calculated, and are shown on the ordinate. Values are presented as the mean ± SEM (*n* = 4). Significant difference in values between the HMF-treated and non-treated cells (** *P* < 0.01, *** *P* < 0.001; Student’s *t* test) and in those indicated by the brackets (^#^ *P* < 0.05, ^##^ *P* < 0.01; Student’s *t* test) are shown.

It is known that neurotrophic factors induce the activation of MEK1/2, which are MAPK kinases that phosphorylate ERK1/2 [26]. We then pretreated the cells with U0126 or PD98059, both of which are inhibitors of the ERK1/2 upstream kinase MEK, 30 min before incubation in the presence of HMF. As shown in Figure 4, the pretreatment of neurons for 30 min with either U0126 (A) or PD98059 (B) significantly abolished the HMF-induced increase in ERK2 phosphorylation. Figure 4 also shows that these inhibitors themselves had no effect on the basal level of the pERK2/ERK2 ratio. These results suggest that HMF could activate MEK1/2, resulting in the phosphorylation of ERK1/2.

**Figure 5:**
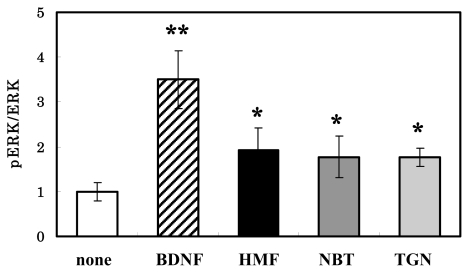
Effects of HMF, nobiletin (NBT) and tangeretin (TGN) on ERK2 activation in rat cortical neurons. Cells were treated with a 50 μM concentration of each compound for 30 min, and harvested for immunoblot analysis using antibodies against phosphorylated ERK1/2 and unphosphorylated ERK1/2. When cells were treated with 50 ng/mL BDNF, the incubation time was 10 min. The density ratio of pERK2 to total ERK2 in untreated cultures was expressed as 1 arbitrary unit. Results represent the mean ± SEM (*n* = 4, different cultures). Significant difference in values between the compound-treated and nontreated cells: * *P* < 0.05; ** *P* < 0.01 (Student’s *t* test).

HMF is one of the *Citrus* polymethoxyflavones, and has 7 methoxy groups. We then examined the effect of its structurally related compounds, NBT and TGN, which have 6 and 5 methoxy groups, respectively. When the neurons were cultured for 30 min with a 50 μM concentration of these compounds, there was no significant difference among them in their phosphorylation-inducing ability (Figure 5). These results indicate that there was no correlation between the number of methoxy residues and the ERK1/2-activating ability of these compounds in neurons. A previous report [7] compared the effect of NBT and its analogues (5-demethylnobiletin, TGN, sinensetin, 6-demethoxy TGN, and 6-demethoxy NBT), which have 5 or fewer methoxy groups, on CRE-dependent transcription and neurite outgrowth in PC12D cells. That study revealed that all of them enhance both CRE-dependent transcription and neurite outgrowth and that NBT, with 6 methoxy groups, shows the most potent activity. These observations indicate that the extent of ERK1/2-activating ability of polymethoxyflavones in neurons was not correlated with that of their neuritegenic activity (neuronal differentiation) observed in PC12D cells.

### 3.4. CREB Activation of Neuron Cultures by HMF

**Figure 6:**
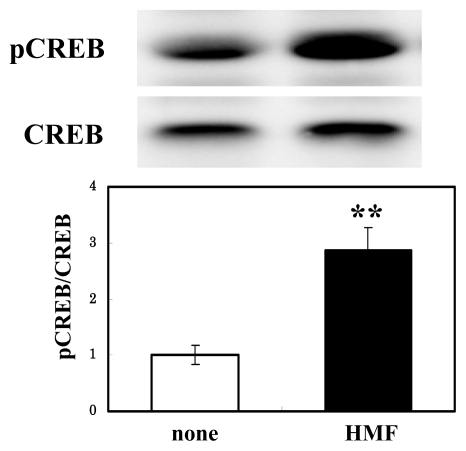
Effects of HMF on cAMP response element-binding protein (CREB) activation in rat cortical neurons. Cells were incubated with or without HMF at the concentration of 100 μM for 30 min, and the cell lysates were prepared and applied to immunoblot analysis. Values are presented as the mean ± SEM (*n* = 4). Significant difference in values between the HMF-treated and non-treated cells: ** *P* < 0.01 (Student’s *t* test).

Among the downstream target of activated ERK1/2, CREB is known to be a critical step in signaling cascade that leads to the structural changes underlying the development of LTP [12]. We thus examined the effect of HMF on the phosphorylation of CREB in neuron cultures. Figure 6 shows that the treatment with 100 μM HMF for 30 min resulted in significant phosphorylation of CREB. Our preliminary examination with PC12 cells showed that HMF enhanced the phosphorylation of the substrates of protein kinase A (PKA, data not shown). These results suggest that, like that of NBT [27,28] and fisetin [4], the effect of HMF might be mediated by the PKA/ERK/CREB signaling pathway.

### 3.5. Enhancement of Memory by HMF

The *in vitro* results mentioned above and the known associations between ERK/CREB activation and memory prompted us to ask whether HMF could improve memory impairment. In the hippocampus, the brain area that plays important roles in long-term memory and spatial navigation, ERK1/2 can be activated through several different signaling pathways implicated in learning and memory, including those involving NMDA receptors and BDNF receptors [29]. It is well established that NMDA receptor antagonists block hippocampal LTP and impair acquisition in the Morris water maze task. In the present study, we thus used the nonselective NMDA receptor antagonist MK-801 to impair learning ability. It was reported that MK-801 at doses higher than 0.1 mg/kg i.p. caused significant motor impairment and severely affected the animals’ ability to complete the water maze task [30]. Therefore, we treated mice with 0.05 mg/kg i.p. of MK-801. This dose of MK-801 did not cause motor weakness and/or sedative effect of mice in this study.

**Figure 7:**
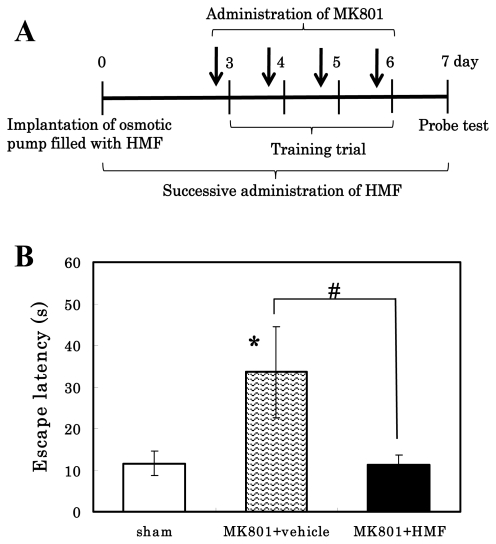
Effects of HMF on deterioration of spatial learning performance in MK-801-treated mice. (**A**) The time schedule of injection of HMF/MK-801 and the MWM test are shown; (**B**) Columns indicate the mean ± SEM (*n* = 5). Significant differences in values between the MK-801-treated and vehicle-treated (* *P* < 0.05; followed by Bonferroni’s Multiple Comparison Test), and HMF-treated (^#^ *P* = 0.05).

Our experimental schedule is shown in Figure 7A. During the training period of the experiments, each mouse was given five trials each day. Consistent with the previous report [30], there was no impairment of swimming speed in MK-801-treated animals. The MK-801-injected mice showed significant spatial reference memory function impairment during the first trial of each training day, but performed significantly better during the latter trial of each day. Also, vehicle- and drug-treated animals showed a reduced escape latency to escape onto the hidden platform with increasing trial periods, because all mice regardless of drug-treatment were able to learn the water maze task.

Figure 7B shows the performance during the first trial of the sixth day of the training period. The MK-801-injected mice (shaded bar) spent more time to get the hidden platform (33.6 ± 11.0 s; * *P* < 0.05) than the sham group (open bar; 11.6 ± 2.94 s), whereas the time to escape of the HMF-treated mice (closed bar) was reduced to that of the sham group (11.3 ± 2.50 s; ^#^ *P* < 0.05). These results indicate that HMF prevented the MK-801-induced impairment of spatial memory.

Twenty-four hours after the training period (Day 7), the hidden platform was removed; and the animals’ ability to remember the location of the platform was then assessed (probe test). During this time, animals spent more time searching for previous location of the hidden platform than for other locations. Neither MK-801 treatment nor HMF treatment significantly affected the time that the animals spent in looking for the previous location of the hidden platform (data not shown). In this study, the mice were not treated with MK-801 immediately before the probe test; and probably the cognition impaired by MK-801 was restored to normal before the start of the probe test, as the effect of MK-801 is transient.

We are now addressing whether HMF can improve brain ischemia-induced learning and memory deficits and whether it can rescue ischemia-induced neuronal death in the hippocampal region. We will show in the near future that HMF, as well as NBT, is beneficial for the treatment of neurological disorders such as Alzheimer’s disease and brain ischemia.

## 4. Conclusions

This study demonstrates that HMF present in *Citrus grandis* (Kawachi bankan) had the ability to induce activation of ERK1/2 and CREB in cultured neurons. The injection of HMF into mice that had been treated with the NMDA receptor antagonist MK-801 restored the MK-801-induced deterioration of spatial learning performance in the Morris water-maze task. These results suggest that HMF, a *Citrus* polymethoxyflavone, is a neurotrophic agent for treating patients with memory disorders, such as Alzheimer’s disease.

## Figures and Tables

**Table 1:** Effect of extracellular signal-regulated kinase (ERK) 1/2 activation by *Citrus* extracts.

Scientific Name	Conventional Name	Activity
*Citrus unshiu*	Unshu mikan	+
*Citrus reticulate*	Imadsu Ponkan	+
*Citrus reticulata*	Mandarin Orange (Kara)	+
*Citrus iyo*	Miyauchi Iyo	−
*Citrus sinensis*	Blood Orange (moro)	+
*Citrus sinensis*	Blood Orange (tarocco)	±
*Citrus grandis*	Kawachi bankan	++
*Citrus depressa*	Hay Flat Lemon	+
0.1% DMSO		−

The neurons were cultured for 30 min in the presence of the ethanol extracts, and then equal amounts of protein were analyzed by immnoblot analysis with antibody against phospho-ERK1/2 along with antibody against the unphosphorylated form of the protein.
